# Recurrent processing improves occluded object recognition and gives rise to perceptual hysteresis

**DOI:** 10.1167/jov.21.13.6

**Published:** 2021-12-14

**Authors:** Markus R. Ernst, Thomas Burwick, Jochen Triesch

**Affiliations:** 1 Frankfurt Institute for Advanced Studies, Frankfurt am Main, Germany; 2 Goethe-Universität Frankfurt, Frankfurt am Main, Germany

**Keywords:** recurrent neural networks, occluded object recognition, recurrent connections, perceptual hysteresis

## Abstract

Over the past decades, object recognition has been predominantly studied and modelled as a feedforward process. This notion was supported by the fast response times in psychophysical and neurophysiological experiments and the recent success of deep feedforward neural networks for object recognition. Recently, however, this prevalent view has shifted and recurrent connectivity in the brain is now believed to contribute significantly to object recognition — especially under challenging conditions, including the recognition of partially occluded objects. Moreover, recurrent dynamics might be the key to understanding perceptual phenomena such as perceptual hysteresis. In this work we investigate if and how artificial neural networks can benefit from recurrent connections. We systematically compare architectures comprised of bottom-up, lateral, and top-down connections. To evaluate the impact of recurrent connections for occluded object recognition, we introduce three stereoscopic occluded object datasets, which span the range from classifying partially occluded hand-written digits to recognizing three-dimensional objects. We find that recurrent architectures perform significantly better than parameter-matched feedforward models. An analysis of the hidden representation of the models suggests that occluders are progressively discounted in later time steps of processing. We demonstrate that feedback can correct the initial misclassifications over time and that the recurrent dynamics lead to perceptual hysteresis. Overall, our results emphasize the importance of recurrent feedback for object recognition in difficult situations.

## Introduction

The primate visual system is capable of recognizing objects with remarkable speed (Potter, 1976; Thorpe et al., 1996). In less than 150 ms, primates can not only correctly classify an object, but also encode visual information in a way that is invariant to scale, translation, and viewing angle (Hung et al., 2005; Isik et al., 2014). Based on this processing speed and the physiological constraints of biological neurons, object recognition in mammals has long been considered to be a mostly feedforward process. The recent success of deep feedforward neural networks in computer vision and machine learning (Krizhevsky et al., 2012; LeCun et al., 2015) lent further credence to this idea. In fact, deep feedforward neural networks have been shown to provide better predictions of neural and behavioral data than previous approaches (Riesenhuber & Poggio, 1999; Serre et al., 2007; Cadieu et al., 2014); Khaligh-Razavi & Kriegeskorte, 2014; Yamins et al., 2014; Rajalingham et al., 2015.

Not unlike the primate visual system, deep convolutional networks use a hierarchy of filters with local receptive fields. However, contrary to their biological counterparts, they lack feedback connections, which are ubiquitous in the ventral visual pathway of primates (Felleman & Van Essen, 1991; Markov et al., 2014). Both anatomical and physiological evidence hint at the importance of recurrent feedback for biological object recognition. Feedback connections were found to be numerous in the visual cortex and may even outnumber feedforward ones (Callaway, 2004; Douglas & Martin, 2004). Furthermore, electrophysiological findings in mammals and humans show that the visual processing of an object unfolds over time, beyond what could be attributed to a pure feedforward process (Sugase et al., 1999; Brincat & Connor, 2006; Cichy et al., 2014). However, a growing number of studies have highlighted the crucial computational role of recurrent connectivity within visual processing (Oram & Richmond, 1999; Mohsenzadeh et al., 2018; Kietzmann et al., 2019; Ernst et al., 2019, 2020; Gwilliams et al., 2020).

The computational advantage of recurrent feedback might be especially prominent for challenging visual input, such as occluded objects. The information about an occluded stimulus is necessarily incomplete and, therefore, prone to being ambiguous. Recurrent processing may “explain away” missing parts of an occluded object (Yuille & Kersten, 2006; Rust & Stocker, 2010) to disambiguate the situation. Specifically, past studies have shown that the recognition of degraded and occluded objects produces delays in behavioral and neural responses, which are believed to be a result of competitive processing within lateral recurrent connections (Johnson & Olshausen, 2005; Adesnik & Scanziani, 2010; Wyatte et al., 2012). For example, object-selective responses have been found to emerge about 50 to 100 ms later for objects that are occluded (Kovacs et al., 1995; Kosai et al., 2014; Fyall et al., 2017) and backward masking procedures, which are believed to interrupt recurrent processing, more gravely impact the recognition of occluded objects compared with unoccluded ones (Wyatte et al., 2012; Tang et al., 2018; Rajaei et al., 2019). In such situations, recurrent connections could complement visual processing by incorporating occluder information (Fyall et al., 2017) or by actively reconstructing information hidden from view (Tang et al., 2014, 2018).

More generally, psychophysical studies on the perception of ambiguous objects have shown that humans' perception often depends on previous experience. This gives rise to perceptual hysteresis and can be demonstrated for auditory and visual stimuli (Brady & Oliva, 2012; Chambers & Pressnitzer, 2014). Recurrent connectivity also seems to mediate this perceptual phenomenon (Kleinschmidt et al., 2002; You et al., 2011).

However, it is less clear whether recurrent connections in artificial neural networks can benefit object recognition in similar ways. Over the last couple of years, there has been a growing body of computational studies addressing this issue. In particular, recent studies have uncovered that introducing recurrent connectivity post hoc can significantly improve performance of feedforward models (Herzog et al., 2020). Spoerer et al. (2020) demonstrated that recurrent connections have the flexibility to dynamically trade speed for accuracy and (Kubilius et al. 2019) developed a neuroscience-inspired recurrent model that performs competitively on ImageNet. With regard to occlusion, past studies could show that recurrent networks indeed perform better on occluded stimuli, but so far the experimental and computational approaches used highly restricted datasets where artificial inputs were only partly faded out or masked (Smith & Muckli, 2010; O'Reilly et al., 2013; Tang et al., 2014, 2018; Spoerer et al., 2017) and more recently (Ernst et al. 2019) and (Kang and Druckmann 2020) indeed use occluder objects, but either do not incorporate class-variability or spatial depth and binocular processing.

Vision is an active process in a three-dimensional (3D) world. Primates perceive occlusions stereoscopically, with two eyes and the visibility of an object is highly dependent on position and viewing angle. For this reason we explored the idea of stereoscopic stimuli and compared the classification performance of recurrent and feedforward architectures (Ernst et al., 2019). However, the objects considered were simplistic sans serif digits, that incorporated perspective cues, but lacked any in-class variability.

Ernst et al. (2020) introduced the first iteration of a dataset called the Occluded Stereo Dataset for Convolutional Architectures with Recurrence (OSCAR). This version consisted of just two components: the occluded stereo MNIST (OS-MNIST) and the occluded stereo YCB (OS-YCB). The use of MNIST digits enabled studying objects with in-class variability for the first time, but target objects were always centered in the middle, only allowing occlusion from the left or the right. Moreover, digits were not downscaled according to distance. The authors reported first evidence that recurrent connections are able to revise wrong first guesses for more sophisticated stimuli. Also introducing images of occluded 3D objects, the contribution was a step towards more natural stimuli, but it lacked an analysis of the representation in the latent space and insights into the evolution of recurrent activity.

In this article, we set out to conduct a thorough and detailed study with novel versions of our OSCAR datasets evaluated on new network models. We compare recurrent and feedforward models on datasets that cover the full spectrum from simple two-dimensional (2D) objects with little in-class variability to real 3D objects including stereoscopic stimuli. We do this to emphasize the generality of our findings and to examine where along this spectrum a potential benefit for recurrent models might disappear. To better understand the benefits and mechanisms of recurrent feedback, we propose a new overall architecture for our models introducing a global average pooling (GAP) operation, that significantly reduces the amount of learnable parameters and enables a quantification and visualization of the effect of recurrent connectivity over time.

Assuming the fundamental structure and naming scheme of (Spoerer et al. 2017) and Liang and Hu (2015), we distinguish bottom-up (B), top-down (T), and lateral (L) connections. Bottom-up connections model the information processing from lower to higher processing regions, while top-down connections model the communication from higher to lower regions. Lateral connections process information within the same region of the simulated ventral visual hierarchy. To test whether recurrent connections benefit classification performance in a natural occlusion scenario, the different models were trained to classify an occluded target object in monocular and stereoscopic input images. Our results show significant performance gains for recurrent networks compared to parameter matched feedforward models. In contrast with earlier works (Ernst et al., 2019, 2020) and in addition to the overall architectural change, we adapt the properties of one of the feedforward networks to more closely match their recurrent counterparts and we add a new, conceptually different, and deeper control model.

Additionally, we investigate how the recurrent feedback shapes the dynamic internal representation of stimuli across time. Representations of occluded stimuli are driven to approximate the representations caused by their unoccluded counterparts. This corroborates the idea that feedback mechanisms can actively “discount” the occluders. Furthermore, we use class-activation mapping (CAM) to demonstrate that recurrent signals can not only revise wrong first guesses, but they are also focusing the network's “attention” on the target such that only informative image regions are used for the final classification. Finally, when tasked with classifying ambiguous stimuli over time, all of our recurrent network models display perceptual hysteresis. To summarize, we make the following four contributions:

(1) We present a significantly enhanced and refined version of our benchmark data set for occluded object recognition called OSCAR v2, to capture the full range of disparity and perspective cues for both natural (handwritten digits) and computer-rendered (3D objects) stimuli.
(2) We test new feedforward and recurrent convolutional network architectures on this data set and present evidence of systematic performance gains for recurrent architectures.
(3) We use CAMs to analyze how recurrent connections enable these networks to focus processing on the target object and “discount” occluders.
(4) We demonstrate and discuss how the recurrent connections give rise to perceptual hysteresis reminiscient of psychological studies.

## Methods

### OSCAR

**Figure 1.**
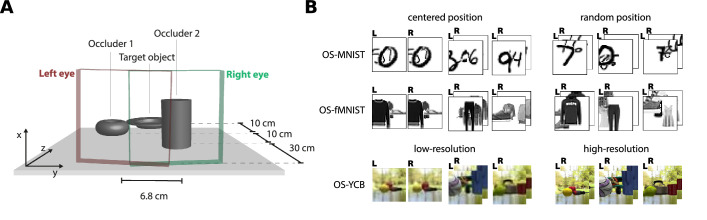
The setup of data generation and the used stimuli. (A) The central object is occluded by two objects arranged into a 3D scene. (B) The stimuli of the new OSCAR version consisting of the three different datasets OS-MNIST, OS-fMNIST, OS-YCB, and their variants.

The OSCAR dataset, first introduced in (Ernst et al. 2020), is composed of stereo images for occluded object recognition. It is intended to bridge the gap between the largely artificial task of recognizing occluded digits (Spoerer et al., 2017) and the natural task of recognizing common 3D objects that are occluding each other. Here, we present version 2.0 of this dataset, which has seen significant improvements and additions. First, we added a third flavor of the dataset called (OS-fMNIST) complementing OS-MNIST and OS-YCB (described elsewhere in this article). Second, we added variants with uncentered-position objects to enforce translational invariance and to make the task more natural and challenging. This way, occlusion can occur to all parts of the target and is not limited to the left or right sides. Third, all the objects across datasets were resized to account for perceived distance and to have a more consistent scale. Finally, OS-YCB now includes high-resolution (320×240 pixel) versions of the images and metadata that enables dedicated training by percentage of occlusion. For an overview of the datasets see Figure 1B. OSCAR v2 is available online together with a dataloader script for PyTorch (https://doi.org/10.5281/zenodo.4085133).

#### OS-MNIST and OS-fMNIST

The OS-MNIST is a novel stereoscopic occluded object dataset loosely inspired by the digit clutter stimuli introduced in (Spoerer et al. 2017). Our dataset progresses one step further toward realistic stimuli by replacing the sans serif digits with samples from the MNIST handwritten database. The in-class variability of MNIST adds additional complexity to our input data, compared with simple digit recognition and encourages the networks to learn a representation that generalizes to different shapes within a particular class.

The dataset comes in two variants, centered position and random position. For the centered position, the target object, that is, the hindmost digit, is kept centered and fixed in the middle of the canvas. Occluding objects are then added sequentially on top. We assumed a distance of 50 cm from the target object to the viewer, and 10 cm less for every added object. The y-axis position of each occluder then is determined by the distance from the viewer along z. Additionally, the size of each added digit is scaled to account for perspective and gives the objects a virtual size. For version 2.0 of this dataset, we decrease the virtual size of the digits resulting in downscaling of digit instances, making the target smaller and preventing upscaling artifacts that might have made occluders more identifiable in the previous version. This procedure creates a pseudo-3D environment with a virtual floor 5 cm below the viewer, on which the objects are standing, making it comparable to the real 3D objects in OS-YCB (discussed elsewhere in this article). The x-axis positions are drawn from a uniform distribution to guarantee images with varying degrees of occlusion.

For the random-position variant, we lift the restrictions of the virtual floor and draw target and occluder positions from a uniform distribution. Nonetheless, we keep the virtual size and scaling according to distance. This, way, objects can be occluded from all sides and angles, making the task more natural and difficult. To create the binocular image-pairs for each scene, the occluders were shifted according to the right parallax given an interocular distance of 6.8 cm. This means that for stereo input, the target-object is always shown at zero disparity. Occluders were chosen in a way that no two instances of one class would appear in the same image.

For OS-fMNIST, we use the same generation procedures and replace the MNIST digits with the Fashion-MNIST clothes objects (Xiao et al., 2017). The OS-fMNIST instances are more extensive covering a larger ratio of the image, which results in a more challenging object recognition scenario.

We created a datasets with 10 occluder combinations per object resulting in 600,000 randomly generated images for training, and 100,000 for testing. All images were rendered at 32×32 pixels. The occlusion percentage of each image is defined as the ratio of occluded pixels to non–occluded pixels of the target object averaged over the two stereo images. Occlusion was constrained to range between 20% and 80% by rejecting everything outside these limits.

#### OS-YCB

To see whether our findings also generalize to a true 3D object scenario, we introduce the OS-YCB dataset. The OS-YCB contains stereo image pairs of 79 common household objects occluding each other. The objects were chosen from the YCB object set, which is an assortment of more than 100 different objects for robotics applications (Calli et al., 2015, 2017). For each image, we placed three virtual 3D objects according to Figure 1A. Analogous to OS-MNIST, the target object is also placed at a distance of 50 cm from the viewer and occluders are placed sequentially 10 cm in front of the last object. In line with the centered-position variants of our other datasets, the target object is kept in the middle of the canvas and occluders are randomly distributed along the *y*-axis. All objects are placed in an upright position and turned by a random yaw angle to provide in-class variability. Objects are placed on a floor 5 cm below the line of sight and a background was chosen to simulate a context with natural image statistics. We repurposed a robotic simulator to serve as a stereoscopic camera. For version 2 of this dataset we use the occlusion percentage metric to divide the dataset into four subsets: 20%, 40%, 60%, and 80% occlusion.

We generated 1,000 images per object for each of the four subsets, resulting in 316,000 stereo image pairs, split 80/20 for training and testing. Stimuli were rendered at 320×240 pixels. For our experiments, we downsample the images to 80×60 and center crop to 32×32 pixels.

### Network models

To evaluate the benefit of recurrent feedback in artificial neural networks we compare a range of two-layer neural networks implemented in PyTorch (Paszke et al., 2019). Following the naming scheme of (Liang and Hu 2015) and (Spoerer et al. 2017), the four possible network types are bottom-up only (B), bottom-up and top-down (BT), bottom-up and lateral (BL), and bottom-up, lateral, and top-down (BLT).

**Figure 2.**
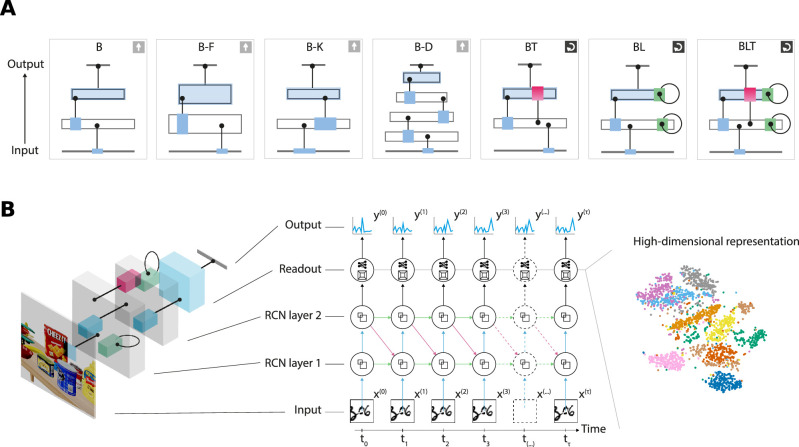
Network overview and details. (A) A sketch of the seven network architectures named after their connectivity. B stands for bottom-up, L for lateral and T for top-down connections. (B) A detailed illustration of the BLT network unfolded for training.

Unless noted otherwise, each model consists of an input layer, two hidden recurrent convolutional layers, and an output layer, see Figure 2A. Convolutional layers with a stride of 1×1 serve as the basis for the bottom-up connections present in all architectures. Following the flow of information the convolved images then go through a 2×2 maxpooling operation with a 2×2 stride, effectively decreasing the dimensionality of the input. Lateral connections are also implemented as convolutional layers with stride of 1×1, whereas top-down connections are transposed-convolutional layers with output stride 2×2 to match the input size one layer below (Zeiler et al., 2010).

As lateral and top-down connections introduce cycles into the computational graph, models using these connections are recurrent neural networks. These networks have internally generated temporal dynamics that set them apart from feedforward networks. Where feedforward networks can be seen as universal function approximators, recurrent neural networks can be thought of as universal dynamical system approximators. To train these networks, we unroll them for a fixed number of time steps and use truncated backpropagation through time (Figure 2B). Unless stated otherwise, we unroll the network structures for four time steps. Thus, the weights for a particular connection within the unrolled network are shared across time. At each time step during training, we feed the same input image into the network and receive a readout from the last layer.

Owing to the surplus of connections, recurrent models have more learnable parameters than their nonrecurrent counterparts for a given number of layers. To address this issue we introduce three additional feedforward network models named B-K, B-F, and B-D. The B-F model doubles the number of convolutional filters from 32 to 64, giving it more features to represent the input data. B-K increases the convolutional kernel sizes from 3×3 to 6×6 compared with the standard B model. The larger kernel of B-K effectively increases the local connectivity of each layer and, thus, we consider it to be the most adequate control model to compare with the recurrent networks. Our third feedforward variant, B-D, adds two more layers of 32 3×3 filters together with the corresponding max-pooling operations in-between. Although this deeper network has the same amount of parameters as the recurrent BL model, its feedforward nature combined with the additional maxpooling layers makes it capable of learning more abstract and potentially more powerful features than all other networks. To provide an additional baseline to our models we also trained a generalized linear model (GLM) on our datasets. This GLM consists of a single fully connected layer with a sigmoid activation function. For a full comparison of model architectures and their numbers of learnable parameters, see Table 1.

**Table 1.** Number of learnable parameters for all models and input channels.

	*B*	*B-F*	*B-K*	*B-D*	*BT*	*BL*	*BLT*	*GLM*
Kernel size	3×3	3×3	6×6	3×3	3×3	3×3	3×3	–
Hidden units	32	64	32	32	32	32	32	–
Layers	2	2	2	4	2	2	2	1
Channels	Number of learnable parameters (OS-MNIST, 10 classes)							

1	9,898	38,218	38,410	28,394	19,146	28,394	37,642	10,250
2	10,186	38,794	39,562	28,682	19,434	28,682	37,930	20,490
Channels	Number of learnable parameters (OS-YCB, 79 classes)							

3	12,751	43,855	40,714	31,247	21,999	31,247	40,495	242,767
6	13,615	45,583	44,170	32,111	22,863	32,111	41,359	485,455

### Recurrent convolutional layer

The central building block of all networks considered here is the recurrent convolutional layer. Each input to one of these layers is denoted by $a_{i,j,k}^{\left(t,l\right)}$, which represents the input from a patch centered on location (i,j) in layer l, computed at time step t of feature map k. Following this notation $a_{i,j,k}^{\left(t,0\right)}$ represents the input stimulus, that is, the image of occluded objects. Before convolution, each input to the layer is batch-normalized to counter covariate shift and speed up learning (Ioffe & Szegedy, 2015; Cooijmans et al., 2017). This technique normalizes an activation a using the mean $\mu_{B}$ and standard deviation $\sigma_{B}$ over a minibatch of activations B.

(1) $$\;\;\;\;\;\;\;\;\;h_{i,j,k}^{\left(t,l\right)}={\mathrm{BN}}_{\gamma ,\beta }\left(a_{i,j,k}^{\left(t,l\right)}\right)=\gamma_{k}^{\left(l\right)}\cdot \frac{a_{i,j,k}^{\left(t,l\right)}-\mu_{B}}{\sigma_{B}}+\beta_{k}^{\left(l\right)},$$

where γ and β are additional learnable parameters. We can then rewrite the batch-normalized output as a vector across all feature maps indexed by k, namely, $h_{i,j}^{\left(t,l\right)}$.

For the feedforward models B, B-K, B-F, and B-D there are no recurrent connections present, thus the preactivation z for a unit in layer l at position (i,j) and time step t can be simply written as:

(2) $$z_{i,j,k}^{\left(t,l\right)}={\left(w_{k}^{(l)B}\right)}^{⊤}h_{i,j}^{\left(t,l-1\right)}+b_{k}^{\left(l\right)},\;\;\;\;\;$$

where t≡0, because feedforward networks cannot be unrolled in time. Here, $w_{k}^{(l)B}$ is the convolutional kernel for bottom-up connections and $b_{k}^{\left(l\right)}$ the bias for feature map k in layer l.

For the BL network, the preactivation gains an additional input owing to the lateral connectivity. This yields:

(3) $$\;\;\;\;\;\;\;\;\;z_{i,j,k}^{\left(t,l\right)}={\left(w_{k}^{(l)B}\right)}^{⊤}h_{i,j}^{\left(t,l-1\right)}+{\left(w_{k}^{(l)L}\right)}^{⊤}h_{i,j}^{\left(t-1,l\right)}+b_{k}^{\left(l\right)},$$

where $w_{k}^{(l)L}$ is the vectorized form of the lateral convolutional kernel and $h_{i,j}^{\left(t-1,l\right)}$ represents the activation from one time step before.

The BT model adds top-down inputs instead of lateral inputs and yields:

(4) $$\;\;\;\;\;\;\;\;\;\;\;\;\;z_{i,j,k}^{\left(t,l\right)}={\left(w_{k}^{(l)B}\right)}^{⊤}h_{i,j}^{\left(t,l-1\right)}+{\left(w_{k}^{(l)T}\right)}^{⊤}h_{i,j}^{\left(t-1,l+1\right)}+b_{k}^{\left(l\right)}$$

for the preactivations. The top-down kernel for the transposed convolution is denoted by $w_{k}^{(l)T}$. Because top-down connections are only received from hidden layers above, the two-layer nature of our models only allows for one top-down connection, compared with two lateral connections.

Adding both lateral and top-down connections to the B architecture yields the BLT model and the corresponding preactivations are computed as:

(5) $$\begin{array}{ccc} z_{i,j,k}^{\left(t,l\right)} & = & {\left(w_{k}^{(l)B}\right)}^{⊤}h_{i,j}^{\left(t,l-1\right)}+{\left(w_{k}^{(l)L}\right)}^{⊤}h_{i,j}^{\left(t-1,l\right)}\;\;\;\;\\ & & +{\left(w_{k}^{(l)T}\right)}^{⊤}h_{i,j}^{\left(t-1,l+1\right)}+b_{k}^{\left(l\right)}. \end{array}$$

Both lateral and top-down connectivity depend on activations from earlier time steps. For t=0, where there would be no previous time step, we set all recurrent inputs to be a tensor of zeros. Following the flow of information for all models the $z_{i,j,k}^{\left(t,l\right)}$ is passed to an activation function (ReLU, $\sigma_{z}$):

(6) $$\sigma_{z}\left(z_{i,j,k}^{\left(t,l\right)}\right)=\max\left(0,z_{i,j,k}^{\left(t,l\right)}\right).\;\;\;\;\;\;\;$$

The final output of a recurrent convolutional layer at time step t then becomes:

(7) $$a_{i,j,k}^{\left(t,l\right)}=\sigma_{z}\left(z_{i,j,k}^{\left(t,l\right)}\right).\;\;\;\;\;\;\;$$

### Network output

After the hidden recurrent convolutional layers the information is passed to a GAP layer, which computes the mean over activations for each feature map. This significantly decreases the amount of learnable parameters compared with the networks in our previous work (Ernst et al., 2019, 2020). These average activations then constitute the input to a fully connected segment with as many output units as there are classes. For classification we use a softmax activation layer, defined as:

(8) $$\begin{array}{c} \text{softmax}{\left(a\right)}_{i}=\frac{\exp(a_{i})}{\sum_{j}\exp\left(a_{j}\right)}.\;\;\;\;\;\; \end{array}$$

The softmax guarantees that the output sums to 1 and can be interpreted as the probability distribution over all possible classes of the dataset.

### Learning

The class memberships of the objects are encoded as one-hot vectors meaning that the target vector y is comprised of elements $y_{i}$ defined as:

(9) $$\begin{array}{c} y_{i}=\left\{\begin{array}{cc} 1 & \text{if}\;i=\tilde{y}\\ 0 & \text{else} \end{array}\right.,\;\; \end{array}$$

where $\tilde{y}$ is the target object label of the image. The cost-function to make the networks' output ${\hat{y}}^{\left(\tau \right)}$ match the target vector y was chosen to be the cross-entropy summed across all time steps τ and all N output units:

(10) $$\begin{array}{ccc} J({\hat{y}}^{\left(0,...\;,\tau -1\right)},y) & = & -\sum_{t=0}^{\tau -1}\sum_{i=0}^{N}y_{i}\cdot \log{\hat{y}}_{i}^{\left(t\right)}\\ & & +\;\left(1-y_{i}\right)\cdot \log\left(1-{\hat{y}}_{i}^{\left(t\right)}\right).\;\;\; \end{array}$$

For stochastic gradient descent, we used the adam optimizer with an initial learning rate of η=0.004 Kingma and Ba (2015). Unless stated otherwise training occurred for 100 epochs with minibatches of size 500. The maximum dynamic learning rate of adam was cut to 10% of its value at epochs 75 and 90. The GPUs for accelerated learning were of type NVIDIA GeForce RTX 2070 SUPER and RTX 2080 Ti. The source code used to define and train all the networks described in the paper is available on github (https://github.com/mrernst/CAR_torch).

### Comparing classification accuracy

As recommended in Dietterich (1998), we use McNemar's test to compare the model performance of two different architectures $f_{a},f_{b}$. McNemar's test (McNemar, 1947) is a statistical test used on paired nominal data. It is applied to a 2×2 contingency table with a dichotomous trait to determine whether the marginal frequencies of row and column are equal. The corresponding test statistic is:

(11) $$\begin{array}{c} \chi^{2}=\frac{{\left(a_{1,2}-a_{2,1}\right)}^{2}}{a_{1,2}+a_{2,1}},\;\; \end{array}$$

where $a_{i,j}$ corresponds with cells in the following four-fold table.

**Table:** 

	
$a_{1,1}$ : number of samples misclassified by both $f_{a}$ and $f_{b}$	$a_{1,2}$ : number of samples misclassified by $f_{a}$ but not $f_{b}$

$a_{2,1}$ : number of samples misclassified by $f_{b}$ but not $f_{a}$	$a_{2,2}$ : number of samples misclassified by neither $f_{a}$ nor $f_{b}$

This methodology does not require repeated training and saves computational resources when evaluating an array of different models. To compare two network architectures with McNemar's test, the same image is classified by both models. Apart from giving the right or wrong answer, the two outcomes can agree with each other or not, resulting in one of the four possible cases of the four-fold table. This procedure is repeated for every image in the test set and thus yields a measure of how different the two networks perform incorporating information about how much the models agree with each other.

To control for the false discovery rate (FDR) when performing all pairwise comparisons of the seven different network architectures, we turn to the following Bonferroni-type correction procedure developed by Benjamini and Hochberg (1995): When testing hypotheses $H_{1},H_{2},...,H_{m}$ based on the corresponding *p*-values $P_{1},P_{2},...,P_{m}$ one shall sort the *p*-values so that $P_{\left(1\right)}\leq P_{\left(2\right)}\leq ...\leq P_{\left(m\right)}$. Let k be the largest i for which holds:

(12) $$\begin{array}{c} P_{\left(i\right)}\leq \frac{i}{m}q^{*},\;\; \end{array}$$

where $q^{*}$ is the level at which the FDR is controlled. All hypotheses $H_{\left(i\right)}$ where i=1,2,...,k are to be rejected. To compare each of our seven different models with each other m=21 hypotheses need to be tested. For the experiments we chose to control the FDR at $q^{*}=.05$.

### Class activation mapping

In contrast with previous works, we significantly decreased the number of learnable parameters using GAP in the output layer of our networks. This also enables us to use a visualization technique known as CAM (Zhou et al., 2016). CAMs arise from training the network architecture itself and do not require any further optimization. Moreover, they have been shown to be suitable for object localization and semantic segmentation (Selvaraju et al., 2020). CAM combines the relative importance of all feature maps of the last layer given the output class and generates a saliency map for each output class. This saliency map represents the importance of each image region for the belief in a specific class. This approach grants a novel perspective on what image regions the network “attends to” during recognition of an object. To statistically compare the evolution of these 2D saliency map distributions we quantify the concentration in two distinct ways. First, we make use of the Gini coefficient,

(13) $$\begin{array}{c} g_{c}=\frac{\sum_{i=1}^{n}2i-n-1}{n\sum_{i=1}^{n}x_{i}},\;\; \end{array}$$

where n is the length of the flattened activation array and $x_{i}$ is the entry at index i. To compare the two distributions across time we use a Kolmogorov-Smirnov two-sample test (Smirnov, 1939) with the Bonferroni procedure by Benjamini and Hochberg (1995), as described before. The nonparametric Kolmogorov-Smirnov test was chosen because the data did not meet the assumption of normality nor the assumption of homogeneity of variance necessary for a *t*-test. To compare four different time steps with each other m=6 hypotheses need to be tested. Second, we use the ground truth segmentation to analyze the sensitivity of CAM activations to the objects being classified. To accomplish this, we assign each pixel of the input image to one of the four categories: background, occluder, overlap, and target. We then compare how much of the total activation mass is captured by an average pixel of each type. To statistically compare the distributions of pixel types within each time step we again use a Kolmogorov-Smirnov two-sample test with Bonferroni correction.

### Perceptual hysteresis

A classic way to illustrate perceptual hysteresis is via viewing a sequence of bistable stimuli. In one approach, the observer sees a series of images in which the stimulus gradually morphs from one class, for example, a man's face to that of another class, for example, a kneeling woman. Subjects at one point perceive a jump in perception from the man's face to the kneeling woman, when watching the sequence of morphed images. However when watching the sequence in the opposite order, the transition between the two ambiguous interpretations occurs for a different image. Thus, the perception of the intermediate images depends on what has just been perceived before. We use a variant of this classic task to investigate whether our networks display a similar hysteresis effect.

**Figure 3.**
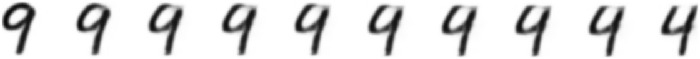
Bistable digit transition The figures from left to right morph from the handwritten digit “9” to the digit “4.” When viewing the pictures from left to right versus right to left, perception can switch from one interpretation to the other at a different image. This reflects perceptual hysteresis.

For our hysteresis analysis, we use unoccluded MNIST as a starting point. We aim to generate a morphing time series between two classes that is ambiguous and bistable during the transition. To create image transitions that fulfill these prerequisites we train a variational autoencoder as described in (Sohn et al. 2015) and then linearly interpolate between hidden representations of the classes. This approach results in 45 morphing time series (see example in Figure 3) with a smooth transition between two different classes. The variational autoencoder is symmetric and has three encoder and decoder layers with 784, 500, and 500 neurons. The latent code is of size 20. It was trained for 100 epochs on MNIST with a batch size of 128 and $\eta =10^{-3}$. We export the resulting images as a time series of size 40. There is no definitive time step where one class supercedes the other, as it is dependent on the representation learned by the autoencoder.

We also test the hysteresis effects for superimposed (blended) MNIST stimuli. To generate the equivalent of the 45 transitions we first calculate the geometric centroid of each class in the high dimensional space generated by the raw images. We then choose the one sample for each class that has the smallest Euclidean distance to the centroid. The resulting ten prototypes are superimposed and linearly cross-faded for 40 time steps.

We train the models BLT, BL, and BT on MNIST without occluders. To ensure stability for longer time series, we unfold the models for 21 time steps instead of just four during training. We use a batch size of 100 and train for 25 epochs. Otherwise we proceed with the same hyperparameters as in the occluded object recognition experiments. For testing we unrolled the networks for the length of the morphing time series and present one image of the time series at each time step.

## Results

### Recurrent connections improve recognition of occluded objects

**Figure 4.**
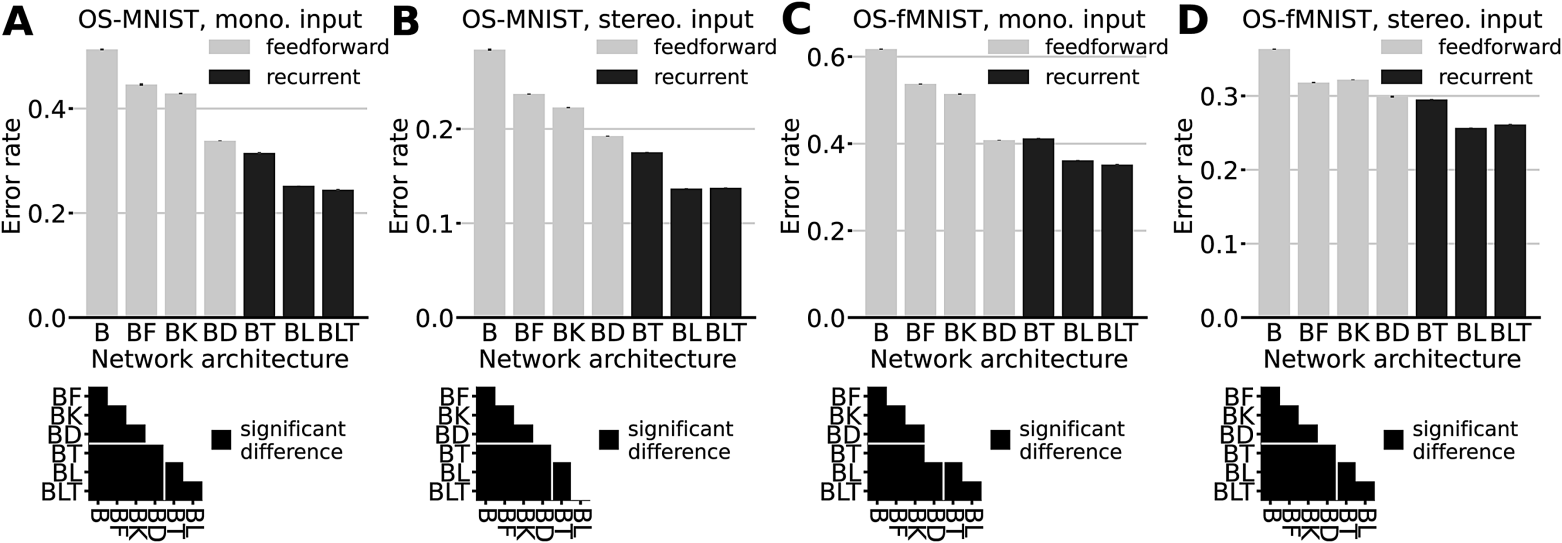
Performance comparison of different network architectures. Error bars indicate the standard error based on five repetitions of the training and testing procedure. Matrices depict results of pairwise two-sided McNemar tests with an FDR of 0.05, black squares indicating significant differences at p<.05. (A) OS-MNIST, mono. input. (B) OS-MNIST, stereo. input. (C) OS-fMNIST, mono. input. (D) OS-fMNIST, stereo. input. Vertical axes are not on the same scale.

To evaluate the benefit of recurrent networks, we trained the seven competing network models to recognize the target objects in our datasets. We compared test performance in the form of classification error (1-accuracy). Figure 4 depicts the error rates for the models trained with the random position variants of OS-MNIST monocular (A), stereoscopic (B) and OS-fMNIST monocular(C), stereoscopic (D). The results indicate that recurrent architectures consistently outperform their feedforward counterparts of near-equal complexity. When evaluated on our novel random position variants of OS-MNIST and OS-fMNIST we observe that all but two pairwise comparisons indicate significant differences (FDR = 0.05).

While B-F have the most parameters of any feedforward model, it only shows a clear advantage over the basic B model for this task. Only for stereo OS-fMNIST B-F displays a higher accuracy than B-K ($\chi^{2}\left(1,N=100.000\right)=3.926,p=.048$). B-K, with its larger 6×6 kernels, performs significantly better in all other cases. Among feedforward networks, B-K is only surpassed by the deeper B-D model with respect to accuracy. Although best among feedforward models, the higher representational power of this deeper network is not enough to significantly outperform any of the recurrent models. The lower 4×3 rectangle in the significance matrix, highlighted by a white line, depicts the comparisons of all feedforward with all recurrent models. The figure shows that in all but one case any of the recurrent networks significantly outperforms any of the feedforward networks. The only exception is for the monocular stimuli of OS-fMNIST (Figure 4C), where the test between B-D and BT did not indicate any significant difference.

When comparing the monocular with the stereoscopic case, we observe consistently lower error rates for the latter. Additionally, the relative performance gain of the recurrent models is consistently higher for stereoscopic input. This is most obvious for the B-D and BT models in OS-fMNIST. While for the monocular case B-D does not perform significantly different ($\chi^{2}\left[1,N=100.000\right]=3.821$, p=.051), for the stereoscopic case BT significantly outperforms B-D ($\chi^{2}\left[1,N=100.000\right]=24.845$, p<.001).

When training on our dataset variants with centered object positions, we observe that the recurrent networks also significantly outperform the three feedforward models B, B-F, and B-K, which we consider the most adequate control model. However, for the centered position task, recurrent models are usually on par or slightly outperformed by B-D. Combined with our results for the novel random position data, we hypothesize that the higher representational power of B-D is responsible for learning a better representation of the particular parts of the objects which are usually left unoccluded owing to the scene arrangement on the virtual floor (in particular the upper middle part). All network weights are initially drawn from a uniform distribution normalized by the kernel size. After training, the mean of weights for bottom-up connections is sometimes positive and sometimes negative depending on the run. For recurrent weights, however, we find that the mean is consistently negative (BLT, mono., layer 1: M=-0.123, SD=0.013, BLT, mono. layer 2: M=-0.129, SD=0.023).

Table 2 contains the model error rates for all the different datasets of OSCAR v2. When trained separately on the four subsets of OS-YCB with varying percentages of occlusion, we observe qualitatively similar patterns (see Appendix A). While the error rates grow with percentage of occlusion, we also see relatively higher performance gains for recurrent models at stereoscopic input. With the exception of B-D, recurrent networks always produce the lowest error rate for all datasets.

**Table 2.** Error rates for different OSCAR v2 datasets and all model architectures.

	OS-MNIST								
	Variant	*B*	*B-F*	*B-K*	*B-D*	*BT*	*BL*	*BLT*	*GLM*
Mono	Centered	.432±.002	.355±.001	.305±.001	.160±.001	.199±.001	.180±.001	.173±.001	.500±.000
	Random	.513±.002	.446±.002	.429±.001	.338±.001	.315±.002	.252±.000	.245±.002	.891±.000
Stereo	Centered	.209±.001	.165±.000	.139±.001	.087±.000	.102±.001	.086±.000	.085±.001	.325±.000
	Random	.284±.001	.237±.001	.223±.001	.192±.000	.175±.000	.137±.000	.138±.000	.887±.000
	OS-fMNIST								
	Variant	*B*	*B-F*	*B-K*	*B-D*	*BT*	*BL*	*BLT*	*GLM*

Mono	Centered	.424±.001	.351±.001	.292±.001	.214±.000	.250±.000	.242±.001	.234±.000	.407±.000
	Random	.617±.001	.537±.001	.514±.002	.408±.000	.412±.001	.362±.001	.352±.002	.895±.000
Stereo	Centered	.260±.001	.225±.000	.209±.001	.177±.000	.203±.001	.190±.000	.195±.000	.303±.000
	Random	.363±.001	.318±.001	.322±.000	.299±.001	.295±.001	.257±.001	.261±.001	.884±.000
	OS-YCB								
	Variant	*B*	*B-F*	*B-K*	*B-D*	*BT*	*BL*	*BLT*	*GLM*

Mono	All	.376±.002	.259±.001	.284±.001	.199±.001	.252±.001	.224±.001	.212±.000	.250±.000
Stereo	All	.166±.001	.092±.001	.105±.001	.064±.000	.090±.001	.071±.001	.069±.000	.091±.000

Standard error based on five independent training runs. Training occurred for 100 epochs, batchsize 500. Best two performances per dataset are highlighted in bold.

### Recurrent connections help to discount occluders

**Figure 5.**
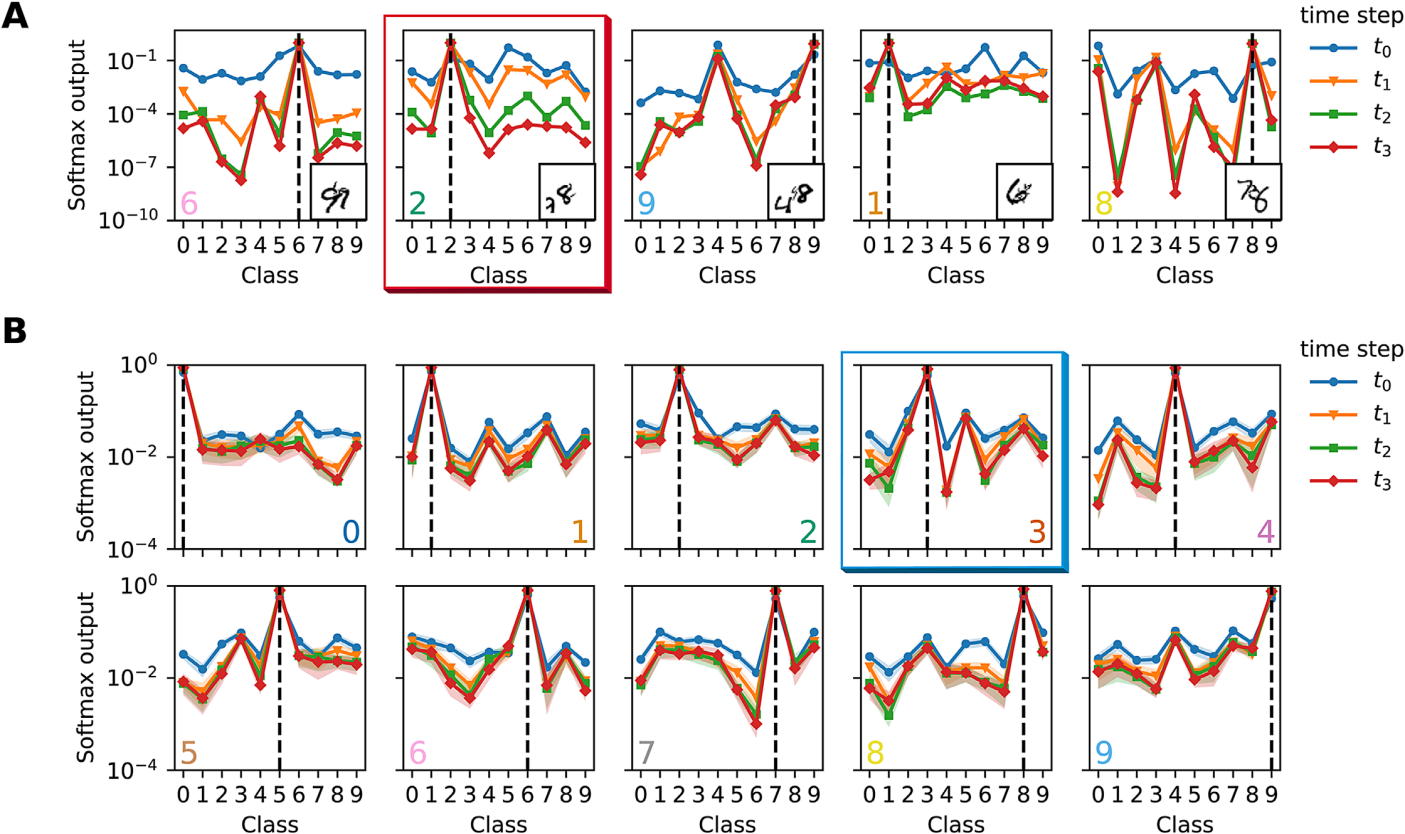
Softmax output of BLT network, trained/tested on OS-MNIST, random position. (A) Specific stimuli illustrating the effect of recurrent feedback. In the example highlighted by a red frame, an incorrect initial guess of “5” (see maximum of blue curve representing the first time step) is corrected to the correct interpretation “2” at the later time steps. (B) Mean softmax output over all test stimuli of all 10 classes revealing systematic reduction of softmax probabilities of nontarget classes with time. The probabilities also reflect systematic visual similarities between different classes such as “3,” “5,” and “8” (see example marked by blue frame). Shaded areas correspond to standard error estimated with a sample size of 10,000 images taken from the test set. Dashed lines and colored digits in the lower corners indicate correct target class. Note the logarithmic scales on the *y*-axes in (A) and (B).

To shed light on the mechanisms behind the improved performance of the recurrent networks, we studied how network activity unfolds over time. For each recurrent model, we obtain a softmax distribution over all possible classes for every time step. The readout can be interpreted as the probability distribution over classes and serves to illustrate how the feedback can revise the models' beliefs over time. Our analysis reveals that correct initial guesses tend to be reinforced, whereas wrong initial guesses are frequently corrected. Figure 5A shows specific examples of this behavior for the BLT network and OS-MNIST (random position).

The shown results are qualitatively very similar when analyzed on BL or BT and when being evaluated on our other occluded datasets. We observe reinforcement of correct first guesses in roughly 80% (panel 1) and revision in 20% (panel 2-5) of the test set images. For example, the second panel from the left shows how the target “2” is initially mistaken for a “5” (blue) and only later is correctly classified.

The softmax activation averaged over all samples of a specific target class is shown in Figure 5B. It reveals that the probabilities assigned to incorrect answers decrease over time. Furthermore, we observe that the network tends to make systematic mistakes at the early time steps which appear to be an expression of visual similarity. For example, the networks often misclassify a “3” as a “5” or an “8” (fourth panel in upper row marked by blue frame) and vice versa.

**Figure 6.**
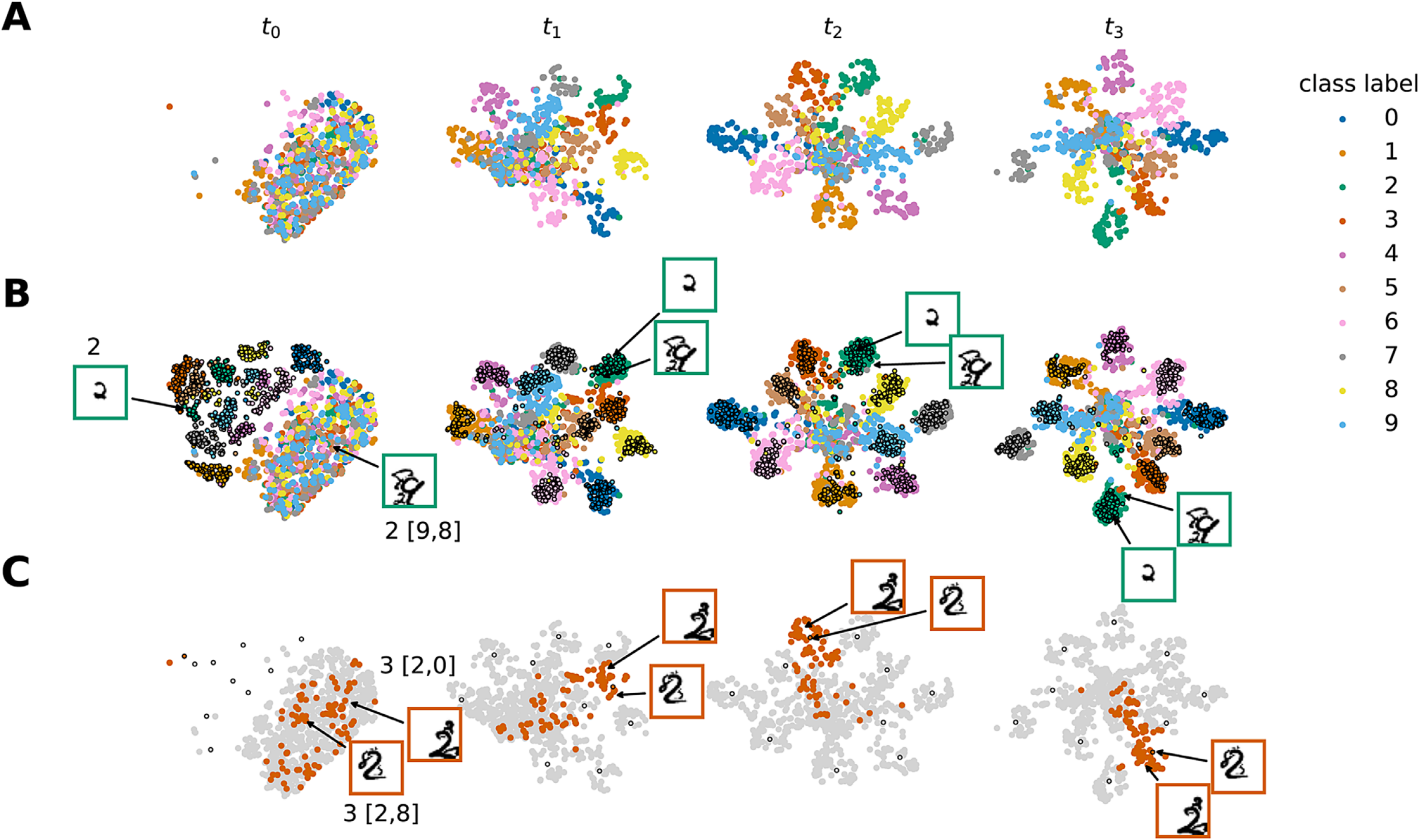
Visualization of the network's internal representation T-SNE depiction of the network's representation of occluded (A) and occluded plus unoccluded (black outline) stimuli (B) evolving in time. (C) highlights the specific class “3” and depicts the geometric centroid of unoccluded classes (black outline), colors represent different classes as shown in the legend.

The softmax output only gives limited insight into the internal dynamics of the recurrent networks. To further investigate how recurrent connections shape the networks' internal representation we consider one layer before the softmax readout. After GAP each input stimulus invokes a 32-dimensional activation pattern $a^{\left(t\right)}$, that changes with time. We visualize this high-dimensional space using t-SNE (Maaten & Hinton, 2008); see Figure 6. The different columns of the figure correspond to the unrolled time steps of the network, while the rows highlight different parts of the test set. Each time step is given to the clustering algorithm separately, thus clusters tend to change their position from time step to time step. The visualization in Figure 6A shows that the internal representations at the first time step are very conflated, but over time become well-separated. Figure 6B includes the representations of unoccluded stimuli (black outline) for all different classes. It is important to note that the network was not trained with this additional data, it was merely given as another test set. The analysis shows that the representations of unoccluded inputs are completely separated from the rest, but are already forming clear clusters for time step $t_{0}$. In contrast, for the occluded stimuli the representations start out rather intermingled but separate over time such that the occluded stimuli come to lie near their unoccluded variants. The inset shows the relative positions of two samples of class “2.” The effect can be seen more clearly when looking at Figure 6C: here, the class “3” is highlighted and we only show the geometric centroid for each unoccluded class representation. The inset pictures show that while two same class pictures are far away in the beginning, they cluster around the average representation of the unoccluded data at later time steps.

**Figure 7.**
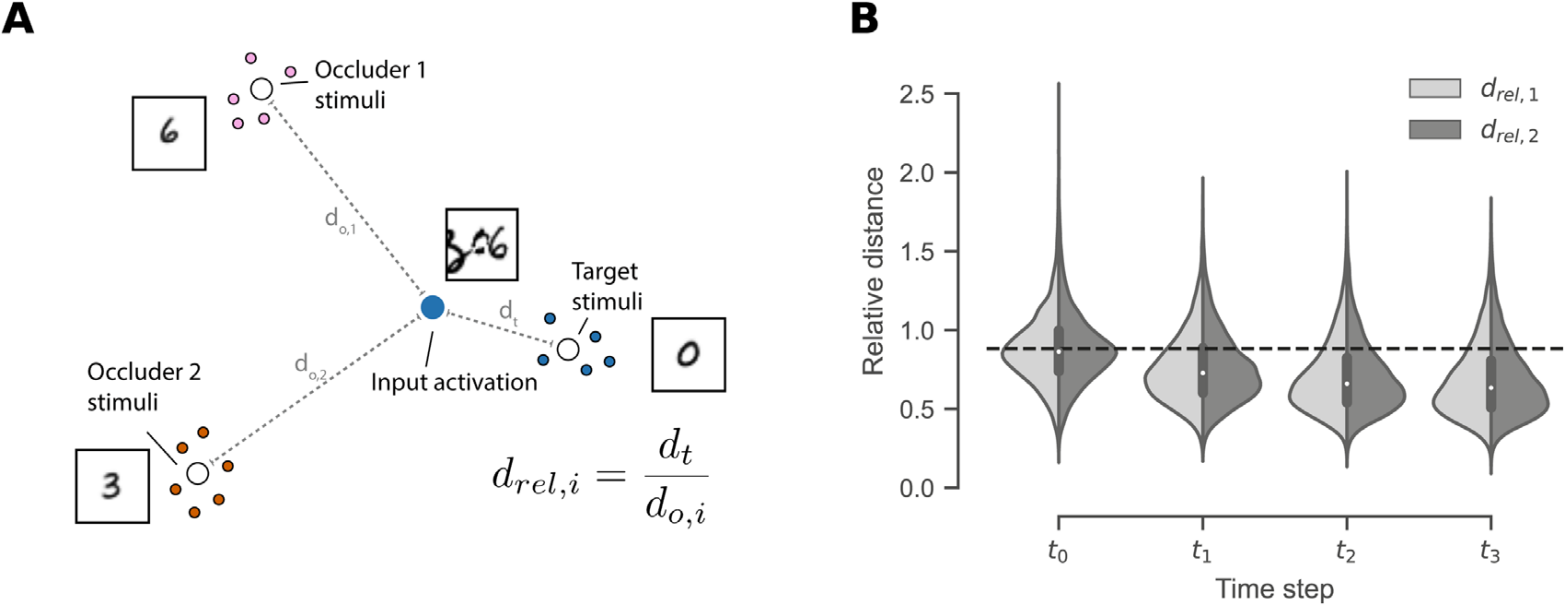
Analysis of the internal representation of occluded stimuli. (A) We define a relative distance measure to quantify if the activation of a stimulus is relatively closer to the centroid of the unoccluded target representations compared to the centroids of the unoccluded occluder stimuli. Values of less than 1 indicate relative proximity to the target. (B) Violin plot displays the relative distances to occluder 1 and 2 at different time steps. Dashed line represents the mean of the distribution at $t_{0}$.

Based on these results, we hypothesize that the recurrent connections are capable of steering the internal representation of an occluded stimulus toward the one of an unoccluded, “pure” stimulus. Thus, we investigate and compare the distances between activations caused by OS-MNIST input and unoccluded input, see Figure 7. We use the Euclidean distance metric extended to the high-dimensional space:

(14) $$\begin{array}{c} {\mathrm{dist}}_{d}\left(x,y\right)=\sum_{i=1}^{d}{\left[{\left(x^{i}-y^{i}\right)}^{2}\right]}^{1/2}.\;\; \end{array}$$

Our analysis considers the relative distances between activation patterns that are invoked by the occluded stimulus, and the centroids of activation patterns caused by unoccluded stimuli corresponding to the target and occluder classes. In contrast with our preliminary analysis concerning stimuli without in-class variability (Ernst et al., 2019), we now examine stimuli that vary in appearance within each class. The result indicates that, over time, the representation of the input approaches that of the unoccluded target class rather than that of the unoccluded occluder classes. The distributions of relative distances for each time step are significantly different (p<.001, one-sided Kolmogorov-Smirnov two-sample test, Bonferroni corrected). These findings provide further evidence that the recurrent feedback allows the network to discount occluders.

**Figure 8.**
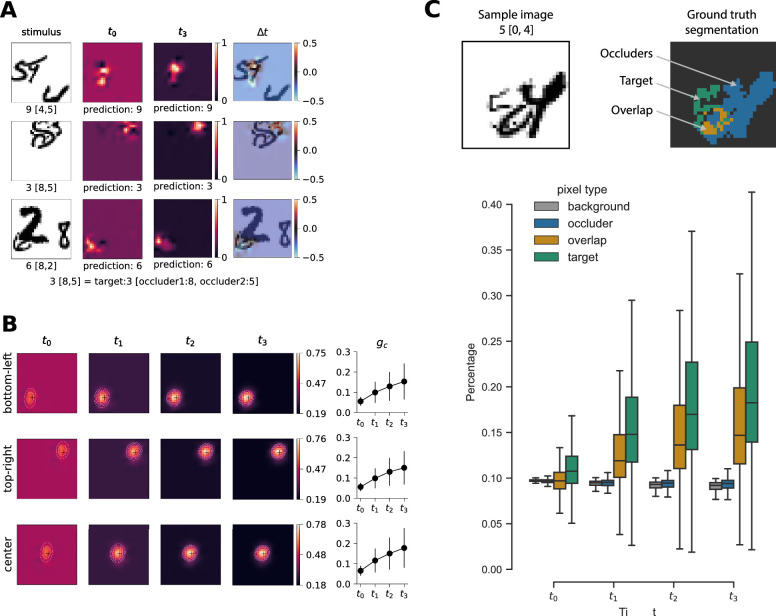
Evolution of CAMs over time, BLT network. (A) Specific samples of OS-MNIST and corresponding CAM. (B) Mean activation map over time for final network prediction. Gaussian fit depicted as four contour lines, crosshairs indicate true position of target. Line plots depict Gini coefficient $g_{c}$ over time, errorbars represent the corresponding standard deviation. (C) Activation mass percentage per pixel for target, occluder, overlap, and background.

To further investigate the effect of recurrent feedback and visualize it in the image domain, we use CAM (Zhou et al., 2016). The CAM illustrates the relative importance of each pixel given the output class. Figure 8A shows one specific example stimulus (OS-MNIST) per row and illustrates the change of CAM through recurrent processing. Most of the time the network correctly classifies the target object even at time step $t_{0}$; however, it attributes a very large area to be important for the classification of that object. Over time, the internal dynamics shape the representation to contract on the specific pixels corresponding to the true target object. Notably, the area of the occluders becomes less important over time as shown by increasingly darker areas at time step $t_{3}$. The panel titled Δt displays the difference of the CAMs from time steps $t_{3}$ and $t_{0}$ with the digits superimposed. It highlights the areas that get amplified and dampened over time relative to the locations of the target object and the occluders.

Figure 8B depicts the mean activation map averaged more than 10,000 test set samples with target objects in three different locations. For all time steps, we display the activation map for the final prediction of the network. Each row shows a test set where the target objects are fixed at a different position on the canvas as indicated by the black crosshairs. Each column shows the evolution over time. The figure illustrates that the network indeed correctly locates the target and shows how the importance assigned to different image locations contracts toward the target location, resembling the network focusing its “attention” on the target.

To better quantify this intuition, we evaluate how concentrated the activations are for each timesteps using the Gini coefficient $g_{c}$. The result of this analysis are shown in the form of line plots on the right. Over time, the concentration of the activation maps rises continuously. All comparisons between time steps are statistically significant (p<.001, two-sided Kolmogorov-Smirnov two-sample test, Bonferroni corrected).

Figure 8C illustrates the second concentration analysis. We divide the input images according to the ground truth segmentation and assess the different pixel types separately. For every time step, we compare each pixel type distribution with each other using a two-sample Kolmogorov-Smirnov test. All comparisons show significant differences (p<.001 after Bonferroni correction). On average a target pixel at $t_{0}$ aggregates M=0.112% of the total activation mass (SD=0.027) and reaches an average percentage of M=0.209% at time step $t_{3}$ (SD=0.103). The general progression of target pixel percentages mirrors the analysis of the Gini coefficient. For $t_{3}$ the target pixel aggregates the most activation mass, followed by the overlap pixel which can be interpreted as part of the target or the occluder (M=0.170, SD=0.083). The background pixel (M=0.090, SD=0.006) holds significantly less mass than the occluder pixel (M=0.093, SD=0.009) and the least mass of all pixel types for $t_{3}$. This general assessment holds true for all time steps. Note that the average occluder and the average background pixel activation mass declines over time. In contrast, the average overlap and target pixel activation mass increases over the same time period.

### Perceptual hysteresis

**Figure 9.**
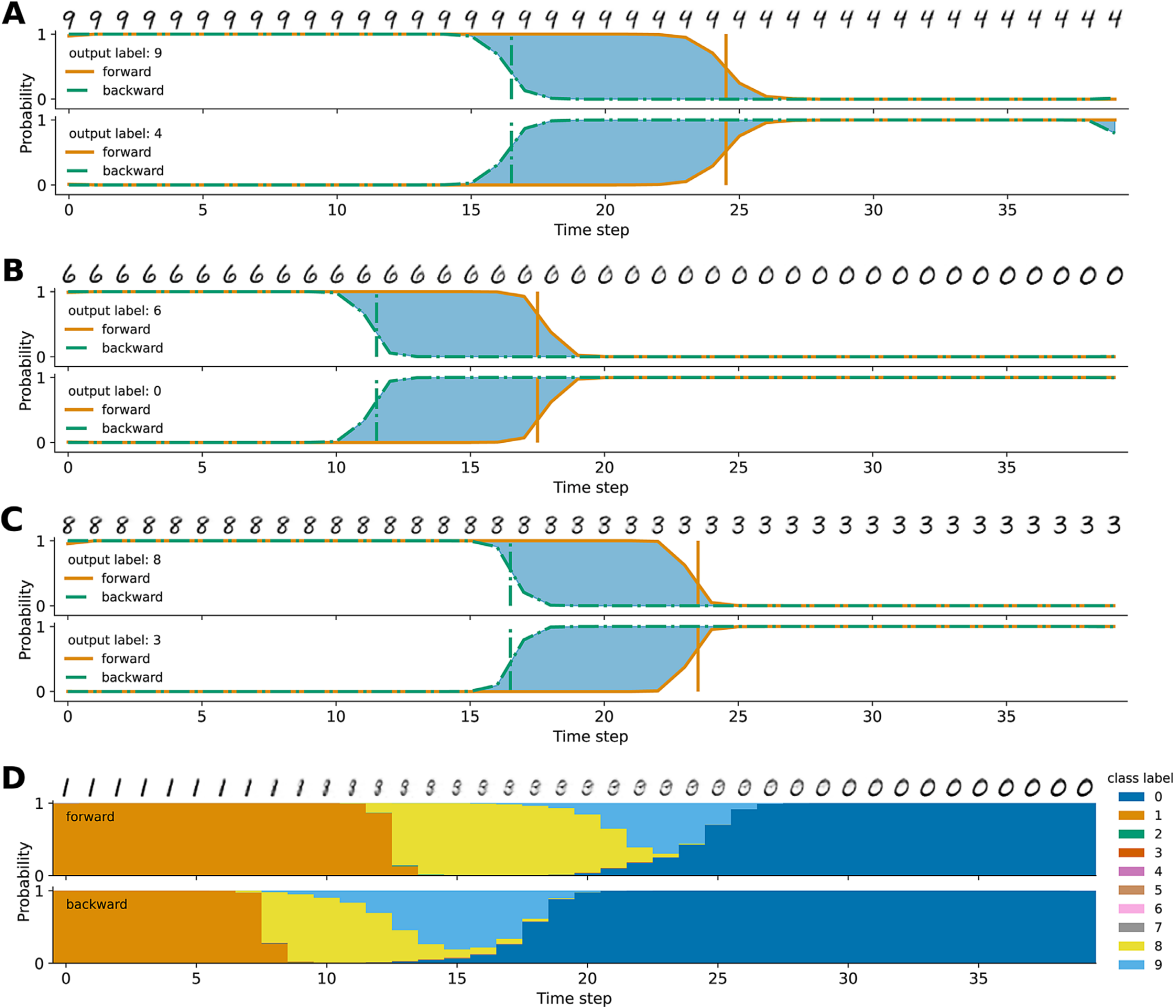
Bistability and perceptual hysteresis, BLT network. Vertical lines correspond with decision boundaries, and blue shaded areas highlight discrepancy in classification between forward and backward sequence. (A) 9↔4, (B) 6↔0, (C) 8↔3. (D) 1↔0, multiple classes involved denoted by color.

So far, our experiments have revealed that recurrent network dynamics unfold over time, altering the output and the hidden representation in significant ways. At any time, the representation of the input does not only depend on the input itself, but also on the state of the network in the previous time step. Thus, the network retains information from one time step to the next. It has an implicit memory trace of the stimulus. We wondered if this implicit memory owing to recurrent processing give rise to perceptual hysteresis as seen in human perception. To answer this question we designed an experiment where we tested the network with sequences of inputs that gradually morph from one class to another and back. Figure 9 shows four representative samples out of the 45 transitions and the softmax output for the two relevant classes along a time series of 40 frames. As can be seen, the decision boundary (vertical lines) between the two classes depends on the inputs from the previous time steps, thus demonstrating hysteresis. This is the only experiment where networks are trained with unoccluded data, that is, the standard MNIST dataset. The training procedure, however, remains the same. Because the recurrent networks BT, BL, BLT have never experienced changing stimuli during training, this experiment shows that the recurrent connections carry important information through time. Additionally, we found that the characteristic curves observed cannot be reproduced by a simple low-pass filtering system that lags behind its input (see Appendix B). We observe these hysteresis effects for all created morphing stimuli, however only approximately 50% of transitions lead to strictly bistable percepts. We define a bistable percept as a forward and backward pass where the model only outputs one of the two relevant classes. To account for the effect of recurrent feedback being able to correct initial guesses, the first and last three time steps are ignored. For percepts that are not bistable, we still observe hysteresis as the forward and backward pass do not yield the same classification. One of these cases is illustrated in Figure 9D, where instead of two, four classes are involved when transitioning between “1” and “0.” For blended transitions we observe qualitatively similar results. Out of 45 transitions, 23 to 26 of the stimuli become bistable percepts for the recurrent networks and hysteresis can be seen for all transitions.

Depending on the ambiguity of the morph, we see stronger or weaker hysteresis in terms of the size of the blue shaded area in Figure 9. We compare the width of the hysteresis curves defined by the number of time steps between the vertical decision boundaries for the different recurrent network types. A one-way ANOVA was performed to compare the effect of recurrent network type on hysteresis curve width. The ANOVA revealed that there was a statistically significant difference in width between at least two groups, F(2,62)=21.222, p<.001. We compared the individual groups post hoc with a *t*-test. The 19 transitions that displayed bistable hysteresis for the BL network (M=9.421, SD=2.160) compared with the 23 transitions of the BLT network (M=5.870, SD=1.191) demonstrated significantly stronger hysteresis, t(40)=6.580, p=<.001. Also, the BT network (M=8.870, SD=2.213) demonstrated significantly larger hysteresis widths than BLT, t(44)=5.6, p<.001. There was no significant effect, t(40)=.793, p=.432, comparing BL with BT.

## Discussion

We investigated whether feedback connections in artificial neural networks can benefit occluded object recognition. Past studies attempted to answer this question using simplistic stimuli which fail to represent the full complexity of natural vision. On the one hand, (Spoerer et al. 2017) used computer rendered digits without any in-class variability. On the other hand, the stimuli used by (O'Reilly et al. 2013) and (Tang et al. 2014, 2018) only blurred out parts of the image rather than introducing occluding objects. More recently, (Kang and Druckmann 2020) tackled in-class variablity, but their test data still lack depth, perspective, and stereoscopic vision.

To overcome these limitations, we have presented a version 2 of our stereoscopic occluded object dataset (OSCAR) that captures the natural variability of object appearance and a range of disparity and perspective cues. With its different subsets (OS-MNIST, OS-fMNIST, and OS-YCB) the dataset bridges the gap from handwritten digit recognition to full 3D object recognition.

We trained a set of seven different network models with and without recurrent connectivity to classify occluded objects. Similar to past studies (Spoerer et al., 2017), but with more naturalistic stimuli, we found that recurrent models reached significantly higher accuracy levels on classification tasks. Additionally, recurrent architectures similar to the ones presented here have been shown to also outperform parameter-matched control models when no occlusion is present (Liang & Hu, 2015), suggesting a rather general benefit of recurrence for object recognition. This is in line with biological observations of how object information in the brain unfolds over time during recognition (Oram & Richmond, 1999; Brincat & Connor, 2006). We cannot completely rule out that the performance gains might disappear when another training regime is used, and the necessity to compare against four feedforward models underscores the challenge to define an appropriate control model. We believe that B-K with its increased local connectivity conceptually matches the recurrent models best. An alternative approach would be to generate a model with the same computational graph as the recurrent model unfolded. However, that would result in a severe mismatch regarding the number of learnable parameters.

Among feedforward models, the deeper architecture B-D performed best. Notably, this deeper network can learn higher order representations than all other networks. We found that B-D can reach comparable performance on centered position data, but not random position data. This suggests that the recurrent networks may have discovered a more general way to reason about occlusions, while the deeper network merely found a way to exploit a bias in the data, which does not generalize to the case of random object positions. The relatively small gap in performance could also be a sign that B-D can approximate recurrent networks due to its additional layers. All other feedforward control models are consistently outperformed by their recurrent counterparts. This includes B-K, which has a larger 6×6 kernel. Within the recurrent ensemble, BT performs worst. The models BL and BLT clearly demonstrate the importance of lateral connections. As lateral feedback is transmitted within one layer and does not have to go through up- and downsampling it may preserve information better.

Evidently, any recurrent computation could also be performed by an appropriately unfolded (and therefore deeper) feedforward network (Liao & Poggio, 2016). This might be one of the reasons why the deeper B-D variant almost reaches the accuracy levels of the recurrent models in our tests. The recurrent network can be viewed as equivalent to such a deeper feedforward version, with certain weights constrained to be identical. Thus, recurrence implies a form of weight sharing in the temporal domain similar to how convolutional layers implement a form of weight sharing in the spatial domain. We speculate that this is the chief reason for the observed performance gains of recurrent networks.

For stereoscopic data, we observe consistently higher accuracy rates than for monocular input. This is most likely due to the fact that stereo input introduces a novel point of view, potentially revealing more information about the target. Additionally, when observed with two eyes, the target is presented at zero disparity, whereas the occluder objects are not. This provides an additional cue regarding what objects are to be ignored. Qualitatively, the results of the statistical network comparisons resemble the ones obtained for monocular stimuli. Interestingly, however, the relative performance difference between recurrent and feedforward models was usually higher for stereoscopic stimuli. This suggests that the recurrent connections are effective in using the additional cues provided by the binocular viewing conditions. Interestingly, during training of the recurrent networks, the sum of both the lateral and the top-down weights became negative on average. This bias towards negative weights might contribute to inhibiting or discounting occluders. As the network's dynamics are governed by the ReLU activation function, a slight bias towards inhibitory weights might also be important to keep activations centered around the nonlinearity and thus facilitate learning.

Our results regarding the performance evaluation are consistent with earlier experiments (Spoerer et al., 2017), but address the issue with more natural image data. The advantages shown for recurrent networks relate to several other interesting phenomena. For random position data it is crucial to assign border ownership at the edge between target and occluders to be able to identify which object is the target before classification. Specialized border ownership cells have been found in the macaque visual cortex that are responsible for this specific task (Zhou et al., 2000). As a common trait, computational models of these cells always incorporate some form of lateral and top-down connections (Zhaoping, 2005; Craft et al., 2007). Thus, it is possible that our recurrent networks also learn some kind of border ownership mechanism to suppress occluders. Such an analysis is left for future work. Interestingly, recurrence has also recently been implicated in the phenomenon of (un)crowding. Crowding describes the phenomenon of an object being harder to perceive when it is presented together with surrounding elements (Bouma, 1973). It is particularly strong in the visual periphery. Doerig et al. (2020) have investigated local versus global processing with deep artificial neural networks and found that feedforward networks cannot explain (un)crowding sufficiently. This is in line with a recent study by (Jastrzębowska et al. 2021), suggesting that recurrent top-down connections might be one of the keys to understanding uncrowding. Thus, there is converging evidence for recurrent processing playing a prominent role in the interpretation of stimuli that are difficult to perceive due to flankers producing crowding or occluders partly hiding the stimulus.

That a recurrent neural network is capable of perceptual hysteresis is not surprising by itself. Nonlinear dynamical systems have long been known to display such behavior. The hysteresis effects we observe are qualitatively similar to experimental results for bistable visual stimuli (Fisher, 1967) and motion perception (Hock et al., 1993), but also see (Stöttinger et al. 2016). However, the functional benefits of hysteresis are still a matter of some debate (Trapp et al., 2021), as well as the impact of psychiatric disorders on hysteresis (Martin et al., 2014). Here we have shown that hysteresis arises in a network that is trained solely for classification. It is not self-evident that optimizing classification performance should lead to hysteresis. Yet, it is consistent with the view of (Poltoratski and Tong 2014) that “hysteresis aids in disambiguating perception during naturalistic visual transitions.” Finally, while the hysteresis typically studied in experiments and exhibited by our network is sometimes referred to as “positive” hysteresis, more recent work has also characterized a “negative” hysteresis, which may be rooted in neuronal adaptation mechanisms and serve a different computational function (Liaci et al., 2018; Sayal et al., 2020). We feel that more work is needed to understand both the mechanisms and functions of these different forms of hysteresis.

In contrast with the majority of work on object recognition, our study considers binocular images and demonstrates clear performance gains for recognition of occluded objects during binocular presentation. A limitation of our approach is our assumption that the target object lies in the plane of fixation, that is, it is seen at zero disparity, while the occluding objects are presented at negative disparities. A more complete model would include a vergence control mechanism that controls the plane of fixation autonomously. Self-calibrating models capable of doing so have been proposed in the active efficient coding framework (Zhao et al., 2012; Eckmann et al., 2020). Another potential avenue for future research is to focus on the stereoscopic networks and analyze whether the individual filters become sensitive to certain disparities that might help to ignore occluders.

We could demonstrate that the recurrent feedback is able to reinforce and even revise first guesses over time. This is in line with the hypothesis that recurrent feedback might “explain away” different alternative hypotheses about the target data (Yuille & Kersten, 2006; Rust & Stocker, 2010). The used loss function consists of a sum over time steps and thus the networks are encouraged to output the correct target at every time step unrolled. However, the structure of the recurrent networks in combination with the input data still seems to favor an iterative convergence to the correct answer. Furthermore, we showed that internal representations of occluded stimuli align with those of unoccluded objects over time and that the networks' internal “attention” focuses on the target object through recurrent processing. We speculate that this focusing may also make recurrent networks more robust against adversarial examples (Goodfellow et al., 2015), but this topic is left for future work.

Another interesting future direction of research would be to investigate the limits of rapid serial visual recognition. Our experiments on perceptual hysteresis illustrate that the recurrent networks' output is generally a function of the current but also previous inputs. In line with the speed–accuracy trade-off investigated by Spoerer et al. (2020) we suspect that this may limit the network's ability to rapidly recognize sequences of distinct inputs similar to how human perception is limited in rapid serial visual presentation and visual masking paradigms.

In conclusion, given the improved performance of recurrent neural network architectures for difficult recognition problems, their greater biological plausibility and their ability to explain various perceptual phenomena, they seem to be the more promising path towards understanding computations in the primate visual system and beyond.

## Figures and Tables

**Figure A1.**
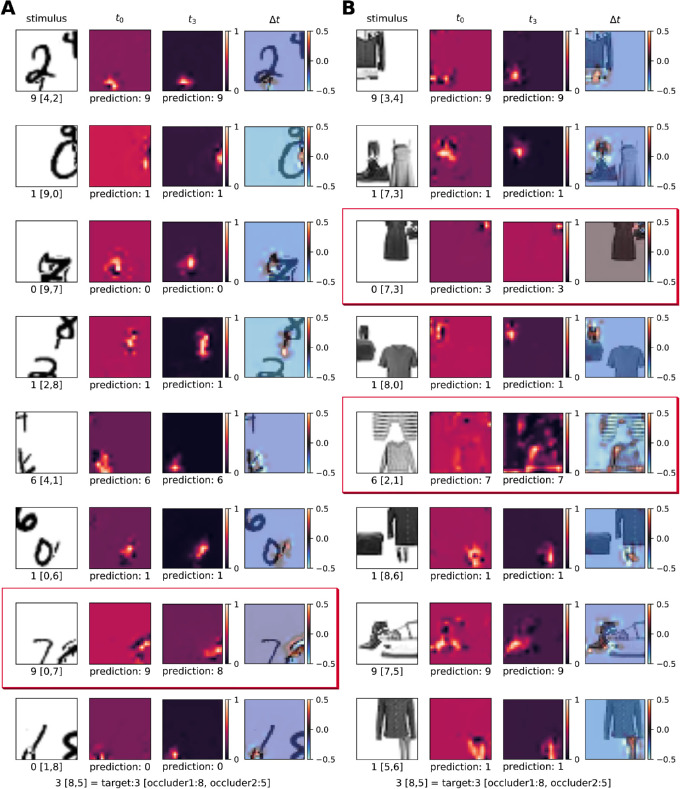
Additional CAMs (A) OS-MNIST, (B) OS-fMNIST. Shown stimuli are randomly sampled from the test set. CAMs are generated by the BLT network. Cases were the network fails to correctly classify the target object are marked by a red frame.

**Figure A2.**
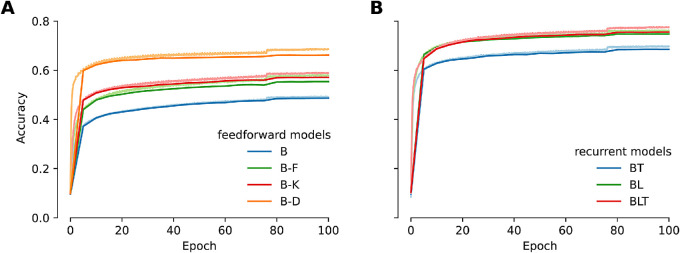
Image classification accuracy across training for different model types. (A) Feedforward models and (B) recurrent models. Solid lines correspond to test accuracy, shaded lines depict training accuracy averaged over five independent runs. OS-MNIST random target, monocular input.

**Figure B1.**
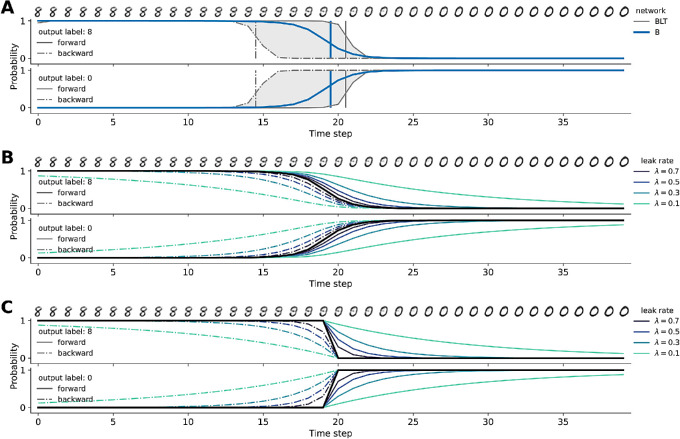
Hysteresis in comparison with a feedforward and a leaky integrator model. (A) Hysteresis curve for the transition 8↔0, similar to Figure 9 (grey), in comparison with a feedforward B network output (blue). (B) Feedforward signal, (C) Step function (black) coupled to a leaky integrator with different time constants λ.

**Figure B2.**
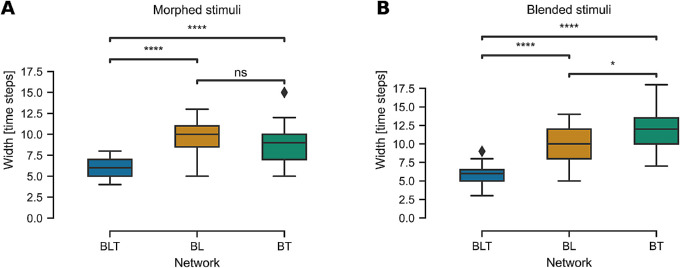
Hysteresis curve width with respect to recurrent models Hysteresis curve width for the three recurrent networks BLT, BL, and BT evaluated on (A) morphed MNIST stimuli (B) blended (linearly superimposed) MNIST stimuli. Distributions were compared using two-sided *t*-tests.

## Appendix A Appendix A: Additional results and figures

As mentioned in the section “Recurrent connections improve recognition performance” we also trained our seven network architectures on subsets of OS-YCB corresponding to different degrees of occlusion. The results of these experiments can be found in Table A3. Qualitatively the results are consistent with the results from training with the whole dataset. The architecture B-D performs best among feedforward models, the architecture BLT performs best among recurrent models.

**Table A1.** Mean and standard deviation of the relative distance $d_{rel}$.

	$t_{0}$		$t_{1}$		$t_{2}$		$t_{3}$	
	M	SD	M	SD	M	SD	M	SD
$d_{rel,1}$	.883	.230	.761	.221	.704	.225	.680	.231
$d_{rel,2}$	.880	.230	.759	.221	.702	.223	.678	.230

Results based on N=10,000 samples. Corresponding analysis shown in Figure 7B.

**Table A2.** Mean and standard deviation of the Gini coefficient $g_{c}$.

	$t_{0}$		$t_{1}$		$t_{2}$		$t_{3}$	
	M	SD	M	SD	M	SD	M	SD
Bottom-left	.055	.021	.099	.052	.129	.071	.153	.089
Top-right	.056	.021	.098	.049	.131	.069	.150	.083
Center	.065	.024	.116	.059	.150	.079	.178	.098

Results based on N=10,000 samples. Corresponding analysis shown in Figure 8B.

**Table A3.** Error rates for subsets of OS-YCB and all model architectures.

	OS-YCB								
	% of occlusion	*B*	*B-F*	*B-K*	*B-D*	*BT*	*BL*	*BLT*	*GLM*
Mono	20	.152±.000	.071±.000	.074±.001	.031±.000	.048±.001	.039±.000	.036±.000	.046±.000
	40	.286±.002	.152±.001	.155±.001	.069±.000	.106±.002	.088±.001	.083±.002	.090±.000
	60	.515±.004	.339±.002	.349±.001	.185±.001	.256±.001	.222±.002	.203±.000	.215±.000
	80	.745±.002	.658±.001	.673±.001	.530±.003	.599±.002	.576±.002	.560±.001	.556±.000
Stereo	20	.072±.001	.039±.001	.042±.001	.016±.000	.025±.000	.019±.000	.019±.000	.033±.000
	40	.140±.001	.068±.001	.075±.002	.031±.001	.051±.000	.041±.001	.038±.001	.052±.000
	60	.260±.003	.137±.002	.146±.002	.072±.001	.104±.000	.085±.001	.083±.001	.095±.001
	80	.433±.003	.279±.002	.285±.002	.167±.002	.213±.001	.182±.001	.175±.001	.198±.001

Standard error based on five independent training runs. Training occurred for 100 epochs, batchsize 500. Best two performances per dataset are highlighted in bold.

**Table A4.** Mean and standard deviation of the activation mass percentage.

	$t_{0}$		$t_{1}$		$t_{2}$		$t_{3}$	
	M	SD	M	SD	M	SD	M	SD
Background	0.097	0.001	0.094	0.004	0.092	0.005	0.090	0.006
Occluder	0.096	0.003	0.094	0.005	0.094	0.007	0.092	0.008
Overlap	0.098	0.018	0.131	0.048	0.154	0.067	0.170	0.083
Target	0.112	0.027	0.161	0.063	0.190	0.087	0.209	0.104

Results based on N=10,000 samples. Corresponding analysis shown in Figure 8C.

To provide a more comprehensive view of the CAM analysis Figure A1 shows eight randomly chosen images from the test sets of (A) OS-MNIST and (B) OS-fMNIST. The discovered focusing mechanism can be seen in the majority of samples. The absence of the effect seems to correspond with cases where the network is unable to correctly identify the target object, highlighted by a red frame.

The networks were implemented in PyTorch version 1.4, training was GPU-accelerated using a single NVIDIA GeForce RTX 2070 SUPER or NVIDIA RTX 2080 Ti. The training of a single network for 100 epochs with intermediate testing every five epochs took 2 to 36 hours depending on the training set, on the network type and GPU used. Figure A2 shows the classification accuracy across training for different models. Test performance saturates and training curves do not diverge much. The first of the two cuts in learning rate is clearly discernible at epoch 75. The depicted learning curves are corresponding to the final error rates of OS-MNIST shown in Figure 4A.

## B Appendix B: Hysteresis and leaky integration

To put our hysteresis results in context, we compare them to the output of a regular feedforward model and a feedforward model whose output has been low-pass filtered using a “leaky integrator.” A leaky integrator can be described by the following differential equation:

(B1) $$\frac{d}{d\tau }x\left(\tau \right)=-\lambda \left(x(\tau )-u(\tau )\right),\;$$

where λ is a leak rate, x is the time-dependent output and, u is the time-dependent input signal. Because our network operates in discrete time steps, we use the discrete time formulation:

(B2) x(t+1)=λ·u(t)+(1-λ)·x(t),t=0,1,2,....

Figure B1 depicts an additional analysis of the hysteresis curves shown in the main text. Figure B1A compares the hysteresis curve produced by the recurrent network BLT (gray) with the output of the feedforward network B (blue). Because the feedforward classifier does not incorporate more than one time step, each stimulus has a definite output. Thus, the curves for the forward and backward passes collapse to a single line. To test whether the hysteresis curve of the recurrent network is a trivial phenomenon that can be replicated by a linear model lagging behind the current input, we augment the feedforward classifier with our leaky integrator model. The results for different leak rates can be seen in Figure B1B. Although this approach does indeed generate different predictions for the forward and the backward pass, the curves significantly differ from what is observed for the recurrent network. For none of the time constants the model replicates the shape of the hysteresis curves of the BLT network. For a comparable hysteresis width of the curve, the leak rate has to be low, which directly corresponds with a very shallow slope. Note that the example shown is taken from the BLT network, which tends to display the smallest width of the hysteresis curves. The leaky integrator model also fails to explain the long uninterrupted stable predictions between the vertical decision boundaries.

**Table B1.** Mean and standard deviation of hysteresis curve width.

	BLT		BL		BT	
	M	SD	M	SD	M	SD
Morphed	5.870	1.191	9.421	2.160	8.870	2.213
Blended	5.741	1.350	9.696	2.422	11.826	2.681

Results based on N=19-26 samples. Corresponding analysis shown in Figure B2.

Figure B1C depicts a more idealized scenario, where instead of the B model we consider the output of a perfect discriminator, that is, a step-function. Here we see the exponential rise and decay that constitutes the solution to the leaky integrator for constant input. Again, the model is incapable of reproducing a curve similar to the observed ones, which suggests that the recurrent network has a persistent memory of the past that stabilizes the internal dynamics for a certain amount of time.

Figure B2 depicts the distributions corresponding to the width of hysteresis curves for different recurrent network architectures. To compare the distributions a one-way ANOVA was performed. The ANOVA revealed that there was a statistically significant difference in hysteresis curve width between at least two groups for morphed stimuli, F(2,62)=21.222, p<.001, as well as for blended stimuli, F(2,70)=48.185, p<.001.
